# Liquid-based biomarkers in breast cancer: looking beyond the blood

**DOI:** 10.1186/s12967-023-04660-z

**Published:** 2023-11-13

**Authors:** You Shuai, Zhonghua Ma, Jie Ju, Tong Wei, Songlin Gao, Yikun Kang, Zixuan Yang, Xue Wang, Jian Yue, Peng Yuan

**Affiliations:** 1 Department of VIP Medical Services, National Cancer Center/National Clinical Research Center for Cancer/Cancer Hospital, Chinese Academy of Medical Sciences and Peking Union Medical College, Beijing, 100021 China; 2 Key Laboratory of Carcinogenesis and Translational Research (Ministry of Education), Department of Endoscopy, Peking University Cancer Hospital & Institute, Beijing, 100142 China

**Keywords:** Circulating tumor cell, Circulating tumor DNA, Circulating tumor RNA, Exosome, Breast cancer, Liquid biopsy, Non-blood biomarker, Cancer diagnosis and therapeutics

## Abstract

In recent decades, using circulating tumor cell (CTC), circulating tumor DNA (ctDNA), circulating tumor RNA (ctRNA), exosomes and etc. as liquid biomarkers has received enormous attention in various tumors, including breast cancer (BC). To date, efforts in the area of liquid biopsy predominantly focus on the analysis of blood-based markers. It is worth noting that the identifications of markers from non-blood sources provide unique advantages beyond the blood and these alternative sources may be of great significance in offering supplementary information in certain settings. Here, we outline the latest advances in the analysis of non-blood biomarkers, predominantly including urine, saliva, cerebrospinal fluid, pleural fluid, stool and etc. The unique advantages of such testings, their current limitations and the appropriate use of non-blood assays and blood assays in different settings are further discussed. Finally, we propose to highlight the challenges of these alternative assays from basic to clinical implementation and explore the areas where more investigations are warranted to elucidate its potential utility.

## Introduction

Breast cancer (BC) has emerged as a major health issue worldwide [1, 2]. For females, BC was the leading cause of cancer incidence in 157 countries and deaths in 119 countries [3]. According to the latest cancer statistics, BC, lung cancer and colorectal cancer(CRC) accounts for 51% of all new diagnosed cases among females in the United States, with BC alone accounting for almost one-third [4]. It is estimated to be 287,850 newly-diagnosed BC cases and 43,250 BC-related deaths in 2022 [4]. Current treatments for BC mainly include surgical procedure, chemotherapy, targeted therapy, radiotherapy, endocrine therapy, immunotherapy and etc. [5–8]. However, a third of BC patients may have relapses, metastasis and chemotherapy resistance, which highlights the importance of promising biomarkers and therapeutic agents to improve early detection and treatment [7, 9]. The explosion in therapeutic approaches for BC could facilitate the development of personalized medicine project.

Recently, liquid biopsy has achieved much attention in recent years [10–13]. It is determined that this promising alternative method exhibits its unique superiority as compared with conventional sampling biopsy, especially for tumor patients that are anatomically hard to sample directly [13–15]. Malignancy in the breast tissue is heterogeneous and broadly classified into different subtypes [16, 17]. In detail, the molecular subtyping of BC relies on the examination of crucial tumor markers through immunohistochemistry tests, such as estrogen receptor(ER), progesterone receptor (PR), HER2, Ki-67 and etc. (Table 1). ER and PR are nuclear steroid receptors that promote the growth of both normal and malignant breast epithelial cells, and their expression is observed in around 75% of breast cancers [18]. Patients who are ER/PR + usually exhibit lower tumor grades, less aggressiveness, and respond to hormone therapy [19]. Around 15% of BC patients exhibit HER2 overexpression, which correlates with aggressive clinical progression and poor prognosis [20]. The remaining 10 to 15% of BC patients are referred to triple-negative breast cancer (TNBC) without positive expression of these three markers, who are typically high-grade and associated with a poorer prognosis [21]. Evidence has highlighted the close association between molecular classifications of BC and patient survival and treatment response, which has been shown in Table 1 and also roundly summarized in other reviews [18, 22]. Recently, the approach of liquid biopsy could also address problems concerning insufficient representativeness [14, 23, 24]. It is highlighted that liquid biopsies enable the repeated collection and longitudinal tracking of dynamic alterations, owing to its feasibility and non-invasiveness [14, 25].

**Table 1:** Breast cancer subtypes category

Breast cancer subtypes	Subcategories	Receptor profile	Subtype prevalence (%)
Hormone positive	Luminal A	ER + or PR + , HER2-, KI67 < 14%	75
	Luminal B	ER + or PR + , HER2-,KI67 > 14% ER + or PR + , HER2 +	
HER2 positive		HER2 +	15
Triple negative breast cancer		ER − , PR − and HER2 −	10–15

**Table 2:** The non-blood Liquid biopsy analytes and potential utility as diagnostic biomarkers

No	Patient	Origin	Technology used	Method of detection	Readout	Function	Refs.
1	49 patients with suspected BCLM	CSF	CellSearch	CTC	The detection of ≥ 1 CSF CTC was associated with a clinical sensitivity of 100% and a specificity of 77.3% for LM diagnosis	Predictina risk of leptomeningeal metastasis	[105]
2	300 patients with 300 EBC and 50 healthy controls	Urine	ddPCR	ctDNA	A higher urinary ctDNA in BC patients. 38% of patients with EBC were detected with mutations who was found to have greater risk of recurrence	Early diagnostic biomarker/Predictina risk of relapse	[112]
3	15 presurgical TNBC	Plasma/urine	Targeted sequencing	cfDNA	A total of 431 shared genetic variants were observed in both body fluids (NF1, CHEK2, KMT2C, PTEN and etc.)	Noninvasive biomarker	[113]
4	1455 patients with EBC and 200 healthy controls	Serum/plasma/urine	ELISA /LC–MS/MS	CTC/ctDNA/TEPs/miRNA/Metabolomics	/	Breast Cancer Biobanking (PBCB) study	[31]
5	250 patients with early BC and 50 healthy controls	Plasma/urine	ddPCR	cfDNA	The cfDNA was observed in plasma and urine measurements, with a sensitivity of 97%, a specificity of 100% and overall concordance of 99%	Noninvasive biomarker	[114]
6	200 BC patients receiving neoadjuvant chemotherapy	Plasma/urine	ddPCR	cfDNA	A strong correlation was affirmed from urinary and plasma DNA at baseline with the correlation coefficient. Patients were divided into two subgroups based on postoperative urine DNA concentration. At 9th months, the HR between the two groups was 1.51	Measure the severity/predict disease relapse	[115]
7	200 patients with BC and 50 healthy controls	Plasma/urine	ddPCR	ctDNA	Index measurements demonstrate over 90% concordance with biopsy. Patients with lower risk of relapse experienced greater declines in detected DNA levels	Predict disease relapse	[116]
8	24 patients with BCLM	CSF	WGS/ddPCR	ctDNA/plasma	ctDNA was detected in all samples derived from patients with BCLM. The suppression of CSF ctDNA was found to be closely associated with better survival time during intrathecal therapy	Predictina risk of leptomeningeal metastasis/Predicts therapeutic response	[97]
9	A HER2-positive mBC patient with brain metastases	Plasma/CSF	WES/ddPCR	ctDNA	Post-treatment ctDNA analysis showed decreased markers level in plasma, consistent with extra-CNS disease control, while increased in the CSF, confirming poor treatment benefit in the CNS	Predicts therapeutic response	[122]
10	24 untreated, primary BC patients and 24 healthy controls	Urine	RT-qPCR	BC-related miRNAs	higher miR-155 levels and lower levels of miR-21, miR-125b, miR-451 in BC patients urine	Noninvasive biomarker	[126]
11	10 breast cancer patients and 10 controls	Saliva	RT-qPCR/WB	Transcriptomes and proteomes	8 saliva-based mRNA (S100A8, CSTA, GRM1, TPT1, GRIKI, H6PD, IGF2BP1, and MDM437) were significantly different between BC and controls. Evaluate the biomarkers in a cohort of 30 breast cancer patients and 63 controls	Early diagnosis biomarker	[127]
12	69 BC patients and 40 healthy controls	Urine	RT-qPCR	exosomal miRNAs	A panel of four urinary microRNA types (miR-424, miR-423, miR-660, and let7-i) as a biomarker tool discriminating BC patients from healthy controls	Early diagnosis biomarker	[134]
13	22 patients with early BC and 26 healthy controls	Urine	RT-qPCR/WB	miR-21/ MMP-1/CD63 in exosomes	MiR-21 expression was significant lower than in the controls. MMP-1/CD63 expression was significantly higher than in controls	Early diagnosis biomarker	[135]
14	16 patients with MPE and 8 patients with non-malignant effusions	Pleural fluid	RT-qPCR	EV-miRNA	miR-1246 was increased, miR-1246 and miR-150-5p were dysregulation in the MPE	Noninvasive biomarker	[138]
15	91 BC patients and 60 controls	Salivary	Biochemical analyses	Biochemical index	Salivary CA125 and sFA levels was higher in patients with BC	diagnosis biomarker( CA125/sFas)	[144]
16	14 patients with MBC	Pleural fluid	WB		The results revealed microvessel formation in the pleura of MBC and the underlying activation of p-JNK/VEGFR2 signaling	p-JNK/VEGFR2 signaling	[145]
17	8 BC cases following chemotherapy	Stool	NMR Spectroscopy	Metabolite	amino acids were found to be upregulated, while lactate and fumaric acids were decreased in patients after treatment	Effect of chemotherapy	[87]

**Fig. 1:**
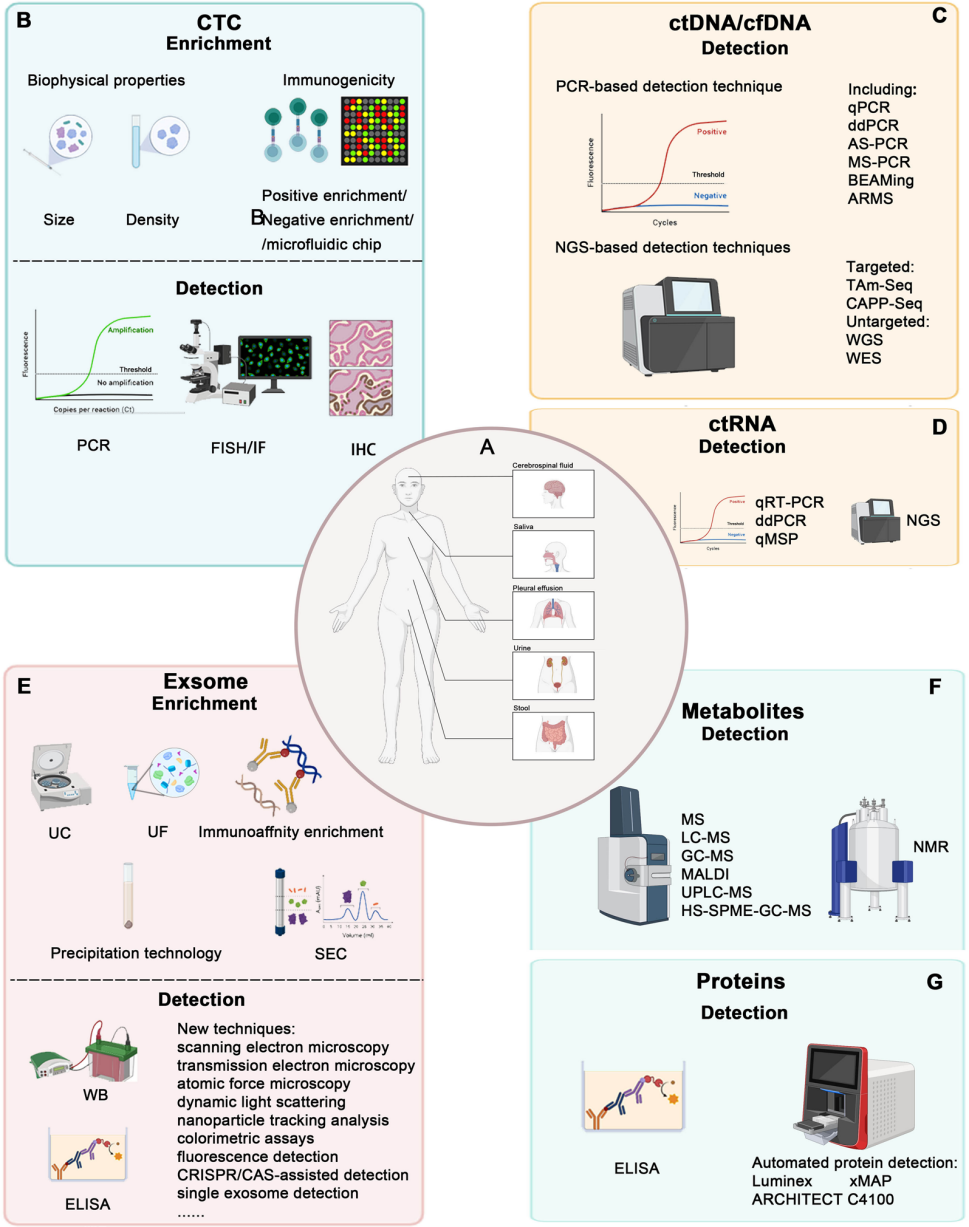
Non-blood sources and techniques of liquid biopsies. **A**. Examples of breast cancer that can be investigated using non-blood sources of ctDNA. **B**–**G**. Techniques for extraction or analysis of liquid biopsy biomarkers in Breast cancer

**Fig. 2:**
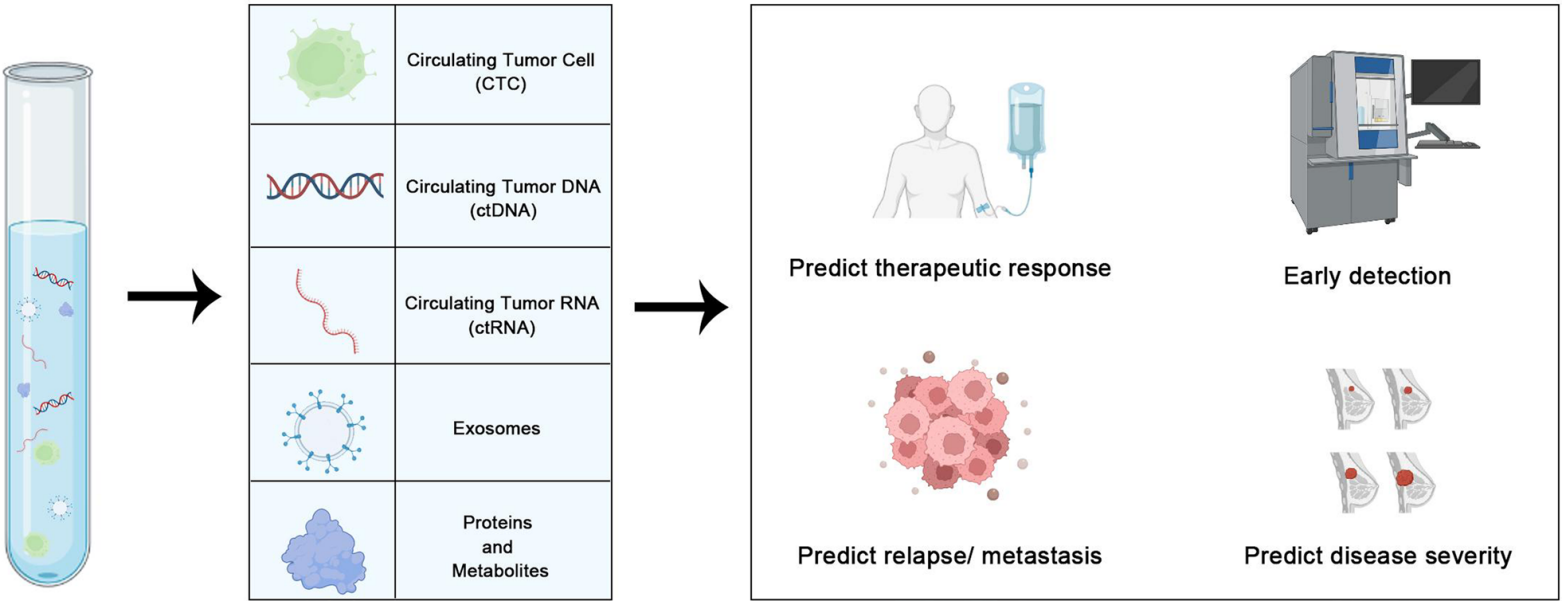
Components and clinical application of non-blood-derived liquid biopsy in breast cancer patients

Emerging evidence has suggested the usage of liquid biopsy in identifying novel biomarkers found within biological fluids, including circulating tumor cell(CTC), circulating tumor DNA (ctDNA), circulating tumor RNA (ctRNA), exosomes, proteins and etc., which could amplify the underlying tumor biology [26–30] (Fig. 1). The advances of liquid biopsies shed new light on the genetic landscape of malignant tumors and pave the way for disease monitoring, treatment efficacy, early detection and prognosis prediction [14]. The implication of this highly-repeatable and relatively non-invasive approach facilitates ongoing tracking of disease progress and seems promising to circumvent tumor heterogeneity [14, 17]. Detecting the dynamics of liquid-based biomarkers is of significant importance in early detection, continuous tracking of the therapeutic efficacy and identification of candidate patients who may exhibit positive therapeutic effects [31] (Fig. 2, Table 2). The identification of the mutation of specific gene, such as those in the BRCA1 or BRCA2, may represent a heightened risk of developing BC, thus identifying individuals who need to be closely monitored [32]. It is worth highlighting the great advancements made in liquid biopsy with the introduction of the Guardant360 CDx platform [33]. It is dedicated to isolating and detecting cancer-specific ctDNA [33, 34]. For BC patients with ESR1-LBD mutations, it has been shown to develop resistance to standard endocrine therapy, resulting in uncontrolled disease proliferation [35]. Most currently, food and drug administration (FDA) has granted approval to Guardant360 CDx for the initial time as a companion diagnostic device for ER + /HER2- metastatic BC with ESR1 mutation [34]. The recent FDA permission for Guardant360 CDx as a groundbreaking blood-based liquid biopsy for diagnosing BC, along with the approval of elacestrant (SERD) for its therapy, signifies a great advancement [34]. Additionally, it has been shown that ctDNA dynamics impact critical functions on monitoring minimal residual disease, providing early indicators of potential disease recurrence, as well as guiding therapeutic strategies. For instance, the determination of HER2 amplification through liquid biopsy could suggest that this group of BC patients may benefit from targeted therapies using drugs like trastuzumab [20, 36]. The continuous acquisition of genetic changes from BC patients may enable the adaptation of treatment strategies as the cancer evolves and the customization of the treatment plan accordingly. Overall, appropriate application of liquid biopsy could capture the unique genetic information to improve early detection and to monitor relapse and responses to treatment, thereby contributing to the development of precision medicine project.

To date, most of the efforts in the area of liquid biopsy focus on the analysis of blood-based biomarkers. Technological advancements also prompt and support the identification and analysis of effective biomarkers from non-blood sources, such as urine, cerebrospinal fluid (CSF), saliva, pleural and peritoneal fluid and stool [37–42]. It is worth noting that the identifications of circulating markers from non-blood sources provide unique advantages beyond the blood and these alternative sources may be of great significance in offering supplementary information in certain settings. In this Review, we outline the latest advancements in liquid biopsy based biomarkers from non-blood biological fluids, and their unique advantages and/or complementary roles in BC. Finally, we also explore the challenges of these alternative assays from basic to clinical use.

## Technologies for liquid biopsies

### Technologies for detecting CTCs

The advances of current technologies enable the identification of CTCs in bodily fluids and improve the sensitivity and precision of detection [43, 44]. The process of CTC determination involves three main steps, including enrichment, detection, and analysis.

The CTC enrichment techniques involve physical and immunomagnetic enrichment. The physical enrichment method can be utilized to separate CTCs based on their physical properties, such as size, density, mechanics etc. [45, 46]. The immunomagnetic enrichment method can be divided into immunomagnetic bead method and immunoadsorption method using a microfluidic chip. Immunomagnetic bead method relies on immunological antibodies, utilizing specific antibodies to bind to antigens on the CTC surface for specific CTC capture [47]. This approach encompasses positive enrichmen using antibodies targeting tumor-related antigens and negative enrichment using antibodies against common leukocyte antigens CD52 [45]. A hallmark of positive enrichment is the CellSearch system, which was the first technology approved by the FDA for CTC detection. It utilizes the principle of immunomagnetic separation, where EpCAM (epithelial cell adhesion molecule) bound to magnetic beads containing specific antibodies [48]. Under the influence of an external magnetic field, this process leads to the diversion of CTCs, achieving their separation and purification. Recent years, several new methods have emerged, such as AdnaTest, MagSweeper and etc. [49, 50]. Negative enrichment strategies for isolating CTCs involve the removal or depletion of blood components other than CTCs, leaving the CTCs behind for subsequent analysis [51]. Microfluidic chip-based enrichment leverages microscale fluid dynamics and engineering principles to effectively capture and isolate CTCs [52]. Devices such as the ‘CTC-Chip’ contain thousands of antibody-labeled microcolumns and have been used to capture CTCs containing specific tumor antigens from LB samples [53].

Technology for CTC detection and analysis include conventional techniques like polymerase chain reaction (PCR) and cell protein detection methods, such as immunofluorescence, immunohistochemistry, and fluorescence in situ hybridization (FISH). Among these, PCR, particularly quantitative real-time PCR, remains the most widely employed technology [54–56].

### Technologies for detecting ctDNA

Owing to the low levels and short half-life of ctDNA/cfDNA in the body, there is an urgent need for sensitive and specific detection methods, which are divided into PCR-based and NGS-based techniques.

PCR is the most widely used method, and techniques based on PCR include standard quantitative PCR (qPCR), digital PCR (dPCR), digital polymerase chain reaction (ddPCR), allele specific PCR (AS-PCR), methylation-specific PCR (MS-PCR), beads, emulsion, amplification, and magnetics (BEAMing), ARMS [57–59]. While PCR-based methods offer higher sensitivity and cost-effectiveness, they have limitations in detecting mutations, driving the advancement of NGS technology.

NGS-based assays are classified into targeted and untargeted categories, including targeted NGS techniques like tagged-amplicon deep sequencing (TAm-Seq) and cancer personalized profiling by deep sequencing (CAPP-Seq). TAm-Seq provides accurate mutation identification and characterization, with the ability to detect DNA levels as low as 2% through primer tagging and genomic sequence analysis [60]. CAPP-Seq technology has high sensitivity and specificity. Among non-small cell lung cancer (NSCLC) patients, the mutational component of ctDNA can be detected with high specificity (approximately 95%) at levels as low as 0.02% [61]. At present, the FDA has granted the approval to two ctDNA testing platforms, Guardant 360 and Foundation One, for identifying genomic alterations in advanced solid malignancies, and their performances utilize NGS to analyze ctDNA/cfDNA dynamics and mutations [34, 62]. Even though targeted ctDNA analysis can detect certain mutations in patients, it cannot detect unknown mutations. Untargeted NGS, such as whole exome sequencing (WES) and whole genome sequencing, have the capability to detect all tumor mutations in patients and facilitate large structural variant detection, as well as whole-genome copy number analysis [63, 64]. Untargeted NGS shows great potential in comprehensively evaluating tumor mutations, but its drawbacks are low sensitivity and high cost.

### Technologies for detecting ctRNA

The detection of ctRNA is typically performed using techniques such as qRT-PCR, ddPCR, methylation-specific quantitative PCR (qMSP), and NGS. Among them, qRT-PCR and ddPCR are employed for verifying gene expression [65, 66]. Notably, methylation level analysis has been applied to ctRNA in the past five years, though yielding less significant results than DNA [67]. NGS provides valuable insights into RNA transcripts, facilitating the understanding concerning tumor heterogeneity and gene expression patterns [68].

### Technologies for detecting exosomes

The distinctive formation and delivery processes of exosomes make their efficient and pure isolation challenging in liquid biopsies. In addition, as tumor exosomes make up only a small fraction of all exosomes in bodily fluids, the detection process also requires high sensitivity and specificity [69].

The separation of exosomes is primarily based on their characteristics, including density, size, surface composition, and exosome precipitation. Several methods have been developed for the separation and detection of exosome proteins and nucleic acids [70]. Ultracentrifugation (UC) is the gold standard for exosome isolation and is the most commonly employed technique, which relies on differences in particle density, shape, and size [71]. Ultrafiltration (UF) is a simple method based on exosome size, offering high purity but limited yield [72]. The combination of UC and UF is now widely adopted, leveraging the advantages of both methods to simplify exosome isolation. Size exclusion chromatography (SEC) is a chromatographic method used for separating molecules in a solution by their size. EVs are washed out from the chromatography column pores ahead of other constituents, thereby separating them from the rest of the sample [73]. The immunomagnetic affinity enrichment uses antibodies targeting tumor-associated proteins (such as CD4, GPC-81, and EpCAM) to differentiate exosomes derived from cancer cells and those from normal cells [71]. Precipitation polymer-based isolation and enrichment of exosomes rely on the use of highly hydrophilic polymers, which reduce the solubility of exosomes and induce their precipitation [74].

Traditional methods for exosome detection is to use WB or ELISA to detect extracellular membrane proteins or other marker proteins. But this methods is complex and not very sensitive [75]. Novel techniques have emerged for exosome detection, such as scanning electron microscopy, transmission electron microscopy, atomic force microscopy, dynamic light scattering, nanoparticle tracking analysis, colorimetric assays, fluorescence detection, CRISPR/CAS-assisted detection, single exosome detection and etc. [76–83].

In conclusion, liquid biopsy is a powerful tool, and the significant advancements in this technology have impacted various aspects of precision oncology, ranging from early diagnosis to the management of advanced and treatment-resistant metastatic diseases.

### Technologies for detecting proteins and metabolites

Human blood and urine contain abundant proteins, and the majority of clinical tests rely on ELISA as the gold standard tool for assessing protein level [84]. ELISA can quantify proteins with relatively high sensitivity and a wide dynamic detection range. However, ELISA requires manual operation using assay kits, and its detection efficiency can not meet the demands of widespread clinical applications [84]. Of note, the establishments of automated protein detection platforms like Luminex xMAP and ARCHITECT C4100 have brought about significant changes, improving efficiency and convenience [85, 86].

It has been commonly believed that cellular metabolites play a significant role in regulating biosynthesis pathways [85, 87]. Currently, the analysis of metabolites is primarily dependent on mass spectrometry (MS) methods, which encompass liquid chromatography-MS (LC–MS), gas chromatography-MS (GC–MS), matrix-assisted laser desorption/ionization-MS (MALDI), and associated techniques like ultra-high-performance LC–MS (UPLC-MS) or headspace solid-phase microextraction coupled with GC–MS (HS–SPME–GC–MS) [88, 89]. MS is the preferred choice for identifying compound structures in complex mixtures due to its ability to accurately measure compound mass and infer chemical composition and structure [89]. MS exhibits remarkable sensitivity and specificity in compound detection [89]. In addition, nuclear magnetic resonance (NMR) is also a commonly used method for metabolite analysis [90]. While NMR exhibits lower sensitivity and specificity compared to MS, it can offer more comprehensive structural information [91].

## CTC

For patients with suspected breast cancer brain metastasis (BrM), the diagnosis relies on the conventional cytology of CSF samples, which was determined as gold standard, and/or MRI imaging [92, 93]. The final diagnosis is based on the determination of tumor cells in the CSF through conventional cytology [92]. Nevertheless, the current method exhibits low sensitivity. As for the conventional cytology of CSF samples, it is worth noting that the efficiency is limited even with the optimal CSF sample volume and analysis timing, with the clinical sensitivity of approximately 45% for 1 CSF sample and 85% for 3 continual CSF samples. It is worth pointing that, despite utilizing the ideal CSF sample volume and timing for assessment, the efficacy is limited, with the clinical sensitivity of roughly 45% for a single CSF sample and 85% for three consecutive CSF sample [94–96]. It was found that repeated samples are required and essential. Moreover, it only allows for the determination of tumor cell presence or absence in CSF rather than a quantitative assessment of the tumor number. Under such scenarios, the use of liquid biopsy sampling could holds considerable importance in verifying the diagnosis of patients with BrM to guide patient management.

Emerging evidence uncovered CSF biomarkers to occult brain metastasis [97–100]. Of note, CSF CTC could function as a promising biomarker to enhance novel diagnostics and assessments of therapeutic outcome, and the achievements of these goals relies on the improvements of novel technologies and devices. The FDA-approved CellSearch^®^ System is a medical device designed for the detection and enumeration of CTCs in peripheral blood. As compared with traditional cytology, CellSearch^®^ System provided superior clinical sensitivity and specificity. In EBC, its CTC detection rates are around 20–30%, whereas in metastatic breast cancer (MBC), detection rates reach 60–70% [48, 101–104]. The identification of CTCs using the CellSearch System is achieved by collecting blood, tagging CTCs, separating them, and detecting those with specific protein markers such as EpCAM. The presence or absence of CTCs in the peripheral blood of MBC patients may suggest therapy effectiveness and replase. In addition, the implication of the CellSearch technique allows for replicable quantification of rare malignant cells and CTC detection in CSF, which provides clinicians with valuable information to assess the medical status of patients, monitor malignant progress, and supply more precise strategies for individuals [48, 101–104].

An interesting study reported by Amelie et al. performed a forward-looking research to uncover CSF CTC in patients with suspected breast cancer leptomeningeal metastases (BCLM) [105]. CSF CTC was detected in all patients, which was diagnosed with BCLM using cytology (n = 18) [105]. It was demonstrated that detecting at least one CTC in CSF corresponded to a sensitivity of 100% and a specificity of 77.3%, which concurred with earlier data and exceeded the performances of CSF cytology [105]. In 40.6% of the involved individuals, CTCs with HER2-positive status were identified in CSF, even in cases initially determined as the HER2-negative (n = 37). These results imply the latent necessity to evaluate the status of HER2 to open up therapeutic opportunities for these patients [105]. Overall, these observations underscore the potential of increased diagnostic sensitivity in detecting LM through the analysis of CSF CTC in liquid biopsy. Moreover, it advocates for the consideration of HER2 status in patients with BCLM to enhance therapeutic options for HER2- patients. Exploring the utilities of CTC in BC clinics may be of great significance.

It is elucidated that CTC determination within blood sources was correlated with therapeutic response in patients with BC [106, 107]. Therefore, additional investigation and comparison between CSF CTC and blood-derived CTC may be interesting and promising, which are required to be further investigated. The detection of CTC using liquid biopsy has revealed significant potential for capturing global features of tumor characteristics, thereby facilitating the precision medicine for patients. However, the available techniques of CTC detection display limitations since the CellSearch system is not totally suitable for CSF samples, with the presence of only few leukocytes. Meanwhile, the amplification of HER2 gene may not be determined by FISH or chromogenic in situ hybridization (CISH) in the context without CTC isolation. These characterizations resulted in the failing of CTC detection in CSF samples. Moreover, the involvement of larger cohort, particularly patients diagnosed with LM, is encouraged in the subsequent research to further comprehensively enrich the understandings of the clinical value of CSF CTC.

## ctDNA

The potential clinical value of DNA within urine has been highlighted over the past several decades [108, 109]. Currently, the advances of using urine has received much attention in the field of liquid biopsies, which could support the non-invasive testing of ctDNA within urine and help overcome current issues correlated with tissue biopsy [110, 111]. Further, the acquisition of urine sample is determined to be simple and convenient, and the presence of medical professionals is often optional and unnecessary. It is notable that the sampling of urine provides much convenience for patients and may be beneficial for early diagnosis, disease evaluation and monitoring, especially under the context of the COVID-19 pandemic.

Recently, an interesting study directed by Gege et al. prospectively explored the function of urinary ctDNA, involving 300 patients with early breast cancer (EBC) and 50 healthy participants [112]. The analysis of baseline index showed that 38% of patients with EBC were detected with either one or both mutations, which can be linked to the presence of minimal residual disease (MRD), and its consistency with tissue biopsy was 97.3% [112]. As compared with healthy controls, a higher urinary ctDNA can be observed in BC patients [112]. For the patients with detectable mutations, it was found to have higher quantities of urinary DNA at 6-month as well as greater risk of recurrence [112]. Moreover, it can be noticed that the researchers also explored and confirmed the stability of urinary DNA, which may improve the accuracy and credibility of the certain research [112]. The utility of urinary DNA was determined as a non-invasive procedure to probe MRD and to conduct real-time monitoring of recurrence in patients with BC. These data revealed that urinary DNA could supplement the existing approaches for monitoring cancer relapse and offering the prospects of early intervention, further highlighting the clinical significance of urinary DNA in patients with BC.

Interestingly, emerging studies have concentrated on the combined analysis of urinary and plasma ctDNA. Henrike et al. investigated plasma-derived and matched urinary DNA samples derived from 15 presurgical triple-negative breast cancer (TNBC) through targeted next-generation sequencing (NGS), thus uncovering the genetic alterations in both body fluids [113]. Combined analysis of both body fluids may supply different profile of TNBC bearing valuable complementary sources, aiding in disease identification and continuous monitoring. In detail, bioinformatic analysis determined 1222 BC-related genetic variants in plasma-derived cfDNA and 2117 variants in urinary cfDNA [113]. A total of 431 shared genetic variants were observed in both bodily fluids, such as the most frequently pathogenic mutated gene NF1, CHEK2, KMT2C, PTEN and etc. [113]. Strikingly, the variant of CHEK2 was determined in all 30 samples, including 15 plasma and 15 urine sample [113]. These observations suggested that urinary cfDNA could be complementary information to plasma-derived cfDNA, and both plasma-derived and urinary cfDNA from TNBC patients are valuable sources to the genetic tumor profile and tumor heterogeneity. Overall, the study directed by Henrike et al. uncovered valuable information of both plasma-derived and urinary cfDNA in a sufficient manner using targeted sequencing techniques and suggested its clinical value as prospective biomarkers for monitoring disease and therapeutic efficacy. However, the participants in this research cohort was limited, which required further involvements to testify the usage of urinary DNA in personalized therapy and enrich the understanding regarding tumor heterogeneity in the future. In a prospective study, 1455 patients suffering from early-stage BC are included, which consent to contribute liquid biopsies to detect ctDNA, CTC, and other profiles every 6 or 12 months for 11 years [31]. Meanwhile, the corresponding data were also required form a control group, which consisted of 200 women aging between 25 and 70 [31]. The identification of effective biomarkers using liquid biopsies enabled clinicians to capture the information of BC patients at the molecular level and to distinguish patients with high risk of relapse, contributing to the establishment of an inter-disciplinary platform for future scientific and clinical studies. Currently, Zuo et al. provided attractive evidence of probing early BC using plasma and urinary circulating cell-free DNA [114]. The effective monitoring of early-stage BC plays an important role in addressing disease relapse. A total of 250 patients with early BC and 50 healthy controls were enrolled in this longitudinal analysis, and a strong agreement of cfDNA was observed in plasma and urine measurements, with a sensitivity of 97%, a specificity of 100% and overall concordance of 99% [114]. Moreover, PIK3CA mutation profiling was also detected in both plasma and urinary cfDNA, and the testing results were compared with the measurements required form tissue samples, with an agreement of 97.2% [114]. The stability and consistency of cfDNA was validated in the analysis of control group, and its continuous measurements of both plasma and urine samples may be of great significance in identifying and monitoring BC [114]. It is worth noting that the declines were determined in both plasma and urinary cfDNA over the six-month serial monitoring, which offer valuable information for physicians to stratifying patients with higher risk of relapse [114]. This study showed a systematic performances of cfDNA in EBC through comparing both sample types, and both types of samples performed well in different aspects. Urine testing exhibited unique advantages of non-invasive nature and accessibility, especially for patients who were hard to get biopsy samples and had resistance to invasive procedures. Overall, the utility of cfDNA was highlighted to be a sensitive and promising approach for identifying high-risk individuals and monitoring disease progress in EBC patients.

Another study reported by Liu et al. also demonstrated good association of urinary and plasma DNA in early BC patients (n = 200) [115]. Moreover, the hazard ratio determined at the 9-month was 1.51 that identified patients at greater risk of relapse and offered valuable chances to monitor dynamic alterations in patients with early BC [115]. The serial measurement of urinary and plasma DNA could enirch the existing testing approaches and supplement the currently-available clinical sources. These data revealed the potential of urinary and plasma DNA as effective biomarker for predicting the disease progression of BC. The study directed by Zhang et al. revealed the significant association between the measurement results of urinary and plasma DNA and disease relapse in patients with BC [116]. The analysis of Receiver operating characteristics curves suggested over 0.95 for both results of urinary and plasma DNA as compared with the controls, uncovering the clinical diagnostic effectiveness of ctDNA derived from blood and urine samples [116]. In addition, the maximum decline in ctDNA level for plasma and urinary ctDNA were 4.0-fold and 6.8-fold, respectively [116]. Patients with lower risk of relapse experienced greater declines in tested DNA levels, suggesting the potential of urinary and plasma DNA in enhancing the prediction of risk [116]. This study reported by Zhang et al. used different approaches of noninvasive testing and uncovered the applicability of ctDNA extracted from blood and urine specimens in the context of clinical diagnostics, risk prediction and disease recurrence in patients with BC [116].

Overall, it is notable that the analysis of and urinary and plasma DNA showed similar features. A larger cohort is required to verify their additional clinical application value. Researchers should attach more importance on investigating urinary DNA for detecting mutation, disease monitoring, and prediction of recurrence. The unique advantages of liquid biopsy could largely contribute to the frequent testing and bring much benefits for patients, especially for the elderly.

Evidence has demonstrated that nearly all deaths for BC are linked to metastasis rather than the primary cancer [117, 118]. BrM has been an increasing clinical issue closely correlated with cancer-related death. Despite this burden, patients with BrM are often disadvantaged by the current diagnostic and therapeutic approaches. Currently, CSF cytology serves as the gold diagnostic test for BrM. The advances of the liquid biopsy may bring much advances for patients with extracranial malignancies, which was expected to function as a promising tool to monitor recurrence, therapeutic response and dynamic alterations rather than replace the gold standard of tumor histology [119–121]. Obtaining tissue samples form patients with BrM is extremely challenging, hard and potentially dangerous. Thus, it is of great significance to uncover the significance of liquid biopsy sampling in primary central nervous system (CNS) disease and possibly secondary spread of CNS.

Recently, an interesting study performed by Amanda et al. investigated ctDNA in the CSF of all 24 patients with BCLM through ultra-low-pass whole genome sequencing, with the purpose of improving the management of patients with relatively poor prognosis [97]. It was indicated that ctDNA was detected in all samples derived from patients with BCLM, regardless of negative cytology or borderline MRI imaging [97]. Conversely, plasma ctDNA was only determined in patients with extracranial disease progression or who had previously received whole brain radiotherapy [97]. These findings suggested the potential of ctDNA marker for timely and accurate diagnosis of BCLM. Importantly, the suppression of CSF ctDNA was found to be closely associated with better survival time during intrathecal therapy, and the rising ctDNA lasted as long as 12 weeks prior to clinical progression [97]. Indeed, a larger sample size was warranted. Further, an adequate panel of control including patients with or without BCLM and patients with nonmalignant brain conditions was required to improve the current study design. Overall, the quantification of ctDNA fraction as an effective biomarker could show great promise in precise diagnosis of BCLM and monitoring of therapeutic response, thereby improving the clinical management and prognostic condition.

Another study reported by Giulia et al. uncovered the genotyping tumor DNA in CSF and plasma of a HER2-positive BC patients with brain metastasis [122]. The droplet digital PCR (ddPCR) and next-generation whole exome sequencing (WES) analysis was implicated to measure dynamic alterations of ctDNA in CSF and plasma, which could represent a minimally invasive and highly sensitive method to identify metastatic tumor [122]. In this clinical scenario, the dynamic alterations of ctDNA level within plasma and CSF yielded more valuable information as compared to the traditional imaging methods for disease monitoring. Post-treatment determination revealed impaired ctDNA level in plasma [122]. Meanwhile, the increase of post-therapy ctDNA was observed in the CSF, which verified limited therapeutic advantage in the CNS [122]. Overall, the certain level of CSF ctDNA corresponded to the changes in tumor burden, illuminating potential advantages in terms of sensitivity over traditional imaging methods. The patients with HER2-positive metastatic BC frequently displayed varying therapeutic response in CNS and non-CNS regions. Analyzing CSF ctDNA may function as a prospective method for tracking disease progression and treatment response, especially in cases of CNS lesions. Utilizing paired liquid biopsies from plasma and CSF may offer precious insights into the optimized management of HER2-positive BC patients with brain metastasis. These findings may be of great significance to identify biomarkers for tracking disease progression and treament response in patients with BrM.

## ctRNA

Previous evidence has demonstrated the emergence of circulating RNA within blood sources [123–125]. It is worth noting that the abundance of ctRNA in non-blood sources of samples from BC patients. A striking study directed by Thalia et al. offered the data of BC-related miRNA levels in urine and determined their diagnostic potential [126]. The analysis results elucidated the four most altered urinary miRNA in BC as compared with controls, including miR-21, miR-125b, miR-155 and miR-451, with an AUC of 0.887 [126]. These observations showed typical expression patterns of miRNAs in the urine of patients with BC, providing support for the clinical potential of urinary miRNA as innovative biomarkers for BC screening. Nonetheless, considering the limited sample size in this pilot study, more extensive investigations were warranted to validate these findings. Another research reported Lei et al. uncovered the role of saliva-based biomarkers in BC detection [127]. A total of 8 saliva-based mRNA biomarkers were identified to be significantly different between BC and control group, including S100A8, CSTA, GRM1, TPT1, GRIKI, H6PD, IGF2BP1, and MDM4 [127]. The accuracy, sensitivity and specificity for the panel of salivary biomarkers was 92%, 83% and 97%, respectively [127]. The evidence provided by Lei et al. affirmed the notion that transcriptomic and proteomic signatures within saliva may serve as diagnostic biomarkers. Salivary biomarkers showed significant discriminative capacity for the detection of BC, characterized by high specificity and sensitivity. These findings may open up the paths for further validation of clinical prediction models in patients with BC [127].

## Exosomes

Exosomes, small extracellular vesicles (30-150nm in size), originate from endosomes formed through the endocytosis of plasma membrane-invaginated endosomes [128]. They are subtypes of extracellular vesicles (EVs) and can be found in numerous human body fluids, including blood, saliva, plasma, breast milk, semen, urine, etc. [70–72]. Exosomes, along with other EVs, play a critical role in intercellular communication. They carry bioactive molecules, including proteins, nucleic acids, lipids and metabolites, and can deliver their content to neighboring cells in a paracrine fashion [128–130].

Within the realm of BC research, exosomes are currently the subject of extensive investigation due to their diverse potential applications in the fields of cancer diagnostics, disease monitoring, prognostic assessment, and therapeutic interventions [128]. For example, certain microRNAs in exosomes have emerged as prospective biomarkers to facilitate the early detection of BC [30, 131]. Furthermore, exosomes are endeavors exploring their utility as nanodrug delivery platforms, serving as vehicles to modulate gene expression within cancer cells or as carriers for anti-cancer agents. Concurrently, exosomes are recognized for their involvement in critical processes of BC progression, including tumor invasion, metastasis, and the orchestration of the tumor microenvironment (TME). The multifaceted utilities of exosomes highlights their importance in BC management. Here, we mainly focus on the involvement of non-blood derived exosomes in BC [132].

Urine represents an alternative source of exosomes [133]. Marc et al. explored the diagnostic capacity of urinary exosomal miRNAs, with 69 BC patients and 40 healthy individuals participated in the research [134]. A panel of four urinary exosomal miRNA (miR-424, miR-423, miR-660, and let7-i) was determined as a set of highly-specific biomarkers through expression level quantification and multilateral statistical assessment, with the sensitivity of 98.6% and specificity of 100% [134]. The diagnosis of BC using urine-based examination of specific exosomal miRNA panels, which showed great promise for effective biomarker, may facilitate the prospective implementation of a non-invasive diagnosis and therapy. Further verification of a series of assays and clinical trials may prompt the introduction of this certain diagnostic method into standard screening practices, offering a favorable non-invasive alternative for the public. Another research directed by Wataru et al. determined miR-21 and matrix metalloproteinase-1(MMP-1) level in urine exosomes from 22 EBC patients without metastasis and 26 healthy controls [135]. The results demonstrated the obvious decrease of miR-21 and significant increase of MMP1/CD63 in BC group compared with control group [135]. This study uncovered that combined expression of miR-21 and MMP1/CD63 in urine exosomes can detect 95% of patients with early BC without metastasis, with the sensitivity of 95% and specificity of 79%. Assessing MMP1/CD63 and miR-21 level within urine exosomes may emerge as an efficient screening method, highlighting the role of miR-21 and MMP1/CD63 as promising biomarkers for early BC detection and treatment [135]. Further studies involving a larger cohort are in an urgent need. It is known that human milk is another source of exosomes, which could be taken up by human intestinal cells in vitro and represent a horizontal information transfer between mother and newborn [136]. An interesting study reported by Oskar et al. uncovered the lncRNAs encapsulated in human breast milk extracellular vesicles (EVs) [137]. It was found that CRNDE, DANCR, GAS5, SRA1 and ZFAS1 were detected in > 90% of EVs from the breast milk samples, which were determined to be crucial regulators and participated in immune cell progress, adipogenesis, and metabolism [137]. The identification of specific lncRNAs within human breastmilk EVs presented a novel mechanism for gaining the understanding of the interaction between the mother and the child, thus impacting critical role on infant development. Besides, evidence suggested that EVs and their contents can also be isolated from pleural fluid, which was a new source and offered access to malignant cells and their microenvironment [138]. Most recently, Samira et al. proposed to compare the expression of EV-miRNA in malignant pleural effusion (MPE) caused by breast (BA-MPE) and lung cancer (LA-MPE), and the effusions induced by heart failure was the control group (HF-PE) [138]. It was shown that the expression level of miR-1246 was obviously increased in the MPE compared with HF-PE group, with the AUC of 0.80 [138]. In addition, between BA-MPE and LA-MPE, miR-1246 and miR-150-5p displayed significant dysregulation [138]. The combination of EV-miRNA was determined as the best classifier in discriminating malignant effusions and controls, with the AUC of 0.81 [138]. These results validated the abundance of EV-miRNAs in pleural fluid from patients with BC or lung cancer using liquid biopsy [138]. However, the underlying mechanism of these markers in tumor progress was not explored in the current research, which determined to be investigated in the future. Moreover, the involvement of large size samples and following-up visits was warranted. The analysis of specific EV-based miRNAs in pleural fluid was highlighted as prospective biomarkers and therapeutic targets. Overall, exploring exosomes from non-blood sources are of great significance to facilitate non-invasive detection and effective therapy.

## Proteins and metabolites

In recent decades, saliva, a type of biological fluid, has been identified to be used in medical assessments [41, 139]. Emerging evidence has highlighted the involvement of saliva as a promising tool to diagnose and monitor disease, as well as guide therapeutics [140, 141]. Its non-invasive characteristics and simple performances contribute to easy obtaining of saliva samples, which requires no needle punctures and largely relieves discomfort for the patients. It is clear that carcinogenesis involves aberrant regulation of various biomarkers and pathways, and correlates with different biological fluids [142, 143]. Overall, non-invasive approaches to improve early detection and treatment is in an urgent need.

Pia et al. uncovered the involvement of salivary biomarkers in diagnosing BC, including 91 consecutive BC patients and 60 controls without neoplastic disease [142, 143]. Cancer antigen 125(CA125) was determined to have anti-adhesive properties, making it an effective marker in BC. Higher salivary CA125 levels of was detected in patients with BC [144]. Specifically, the mean salivary CA125 concentration was 102.1 pg/ml in the control group and 267.6pg/ml in the BC group (p < 0.01) [144]. Further, the soluble Fas (sFas) concentration in turn was 84.1 pg/ml and 145.9 pg/ml, respectively (p < 0.01) [144]. The area under the ROC curve was 0.68 for CA125 (95% CI 0.05–0.56) and 0.67 for sFas (95% CI 0.08–0.55) [144]. In the saliva of patients with BC, CA125 and sFas showed significant increase, indicating their prospective clinical use as diagnostic tool to distinguish BC patients with healthy individuals. However, the casual relationship between study variables was not established in this cross-sectional design. The involvements of different populations could help to verify the obtained results since all participants were from the southern Spain, thus further confirming the role of representative salivary markers in BC.

These is no consensus as to which types of markers provide the best performances in precise diagnosis and treatment, such as proteins, metabolites, and etc. Zhang et al. uncovered BC discriminatory biomarkers in saliva using denovo discovery and validation approaches. A total of 8 abnormally upregulated mRNA biomarkers and 1 protein candidate were screened out through combined analysis of microarray, proteomic profiling and qRT-PCR assays, including S100A8, CSTA, GRM1, TPT1, GRIKI, H6PD, IGF2BP1, and MDM4 [127]. The salivary biomarkers were validated in 30 BC individuals and 63 controls, and not affected by confounding factors, with an accuracy of 92%, sensitivity of 83% and specificity of 97% [127]. These data revealed that the identified salivary biomarkers exhibited strong discriminatory potential in diagnosing patients with BC, which further enriched the understanding of early detection of BC. More explorations of the underlying mechanism of these salivary biomarkers was warranted.

Malignant pleural effusion is a common complication in metastatic breast cancer (MBC) and its accumulation was associated with an ongoing angiogenic process, enhanced vascular permeability and pleural inflammation. A current study performed by Chih et al. determined the angiogenic role of breast cancer-associated pleural fluid (BAPF) on endothelial proliferation, angiogenesis and migration. Chih et al. collected BAPF from 14 patients with MBC, which was cultured with HUVECs to recapitulate the molecular alterations in subpleural endothelial cells [145]. It is reported that the malignant progression of TNBC is much more aggressive as compared with hormone receptor-positive breast cancer (HPBC) [145]. Here, it was found that both BAPF-HP and BAPF-TN exclusively activated JNK signaling among all MAPKs in HUVECs [145]. These findings elucidated the role of subpleural endothelium in promoting tumor metastasis. Current evidence has highlighted the promising role of JNK pathways in TNBC animal models [146]. The elevated expression of VEGFR1 and VEGFR2 was observed in HUVECs cultured with BAPF. Interestingly, the usage of VEGFR2 inhibitor could impair angiogenesis induced by BAPF, which could be used to develop a therapeutic strategy for MBC complicated with malignant pleural effusion (MPE) and lay the groundwork for future therapy for MBC based on hormone receptor status[145]. Recently, metabolites has arisen as promising biomarkers to monitor disease prognosis and therapeutic response. The assessment of stool provides a non-invasive approach to reveal the dynamic changes of metabolites, which leads to more specific and sensitive understandings. Oumaima et al. represents the first study of metabolic dynamics of stool from BC cases following chemotherapy (n = 8) [145]. It was found that the fecal metabolome signature was dramatically altered in BC patients following two and three cycles of chemotherapy as compared with patients undergoing the first cycle or without treatment [145]. Specifically, amino acids were found to be upregulated, while lactate and fumaric acids were decreased in patients under the second and third cycles compared with patients before treatment [87]. The alterations of the short-chain fatty acids (SCFAs) were detected to be more noticeable and tended to increase from the second treatment cycle. Identifying the particular fecal metabolic profiles that mirror the biochemical alterations occurring throughout the chemotherapy is of great clinical importance, as it emerges as novel means for tracking the status of patients with BC, offering valuable information to clinicans to enhance the treatment management. However, the number involved in the current study was low, which requires a larger cohort. Moreover, the implication of metagenomic analysis may be of great significance to better enrich the knowledge regarding the correlation between therapeutic response and different bacteria. Overall, these observations constitutes the first step of metabolites implicated in patients with BC, which shed new light on BC monitoring and therapy.

## The role of blood-based liquid biopsies and its comparison with non-blood liquid biopsies

Recently, blood-based liquid biopsies have been extensively used in the detection of various cancers, including BC [15, 123]. This advanced method enables the continuous and relatively non-invasive acquisition of important genetic information, facilitating the tracking of disease progress and therapy efficacy and guiding personalized treatment strategies [106]. Moreover, the identifications of blood-based biomarkers through liquid biopsy shed new light on BC diagnosis and prognosis prediction [106].

Of note, blood-based liquid biopsies have been shown to exhibit certain advantages over non-blood based liquid biopsies. Firstly, the advances of commercial reagent kits available for the extraction of substances from plasma and established standardized procedures of blood-based liquid biopsies prompts detection and analysis of research targets [33, 40]. Furthermore, when compared to certain non-blood bodily fluids such as CSF and pleural effusion, blood collection is minimally invasive and allows for longitudinal sampling at different time intervals. Meanwhile, blood-based liquid biopsies also show disadvantages as compared with non-blood based liquid biopsies. For specific tumor types or anatomical locations, liquid biopsies based on non blood sources may demonstrate greater sensitivity than those relying on blood sources. For example, saliva can directly interact with oropharyngeal cancer, and stools are in close proximity to colorectal diseases [39, 147, 148]. In comparison to liquid biopsies from blood, both of saliva and stools exhibit higher detection rates [39, 147]. Similarly, urine-based liquid biopsies might offer increased sensitivity when diagnosing renal cell carcinoma and urinary tract epithelial cancer [149]. It is worth highlighting that blood-based liquid biopsies have limited representation of CNS disease. In contrast, CSF is less affected by clonal hematopoiesis, allowing for the detection of mutation at high variant allele frequencies. This make CSF-based liquid biopsy an appropriate choice for diagnosing BC patients with brain metastasis or other CNS disease.

In different situations, both blood-based and non-blood-based liquid biopsies come with distinct pros and cons. Adapting testing methods to specific needs can optimize patient benefits. The combined use of blood-based and non-blood-based liquid biopsy techniques may complement each other and provide a more comprehensive landscape of tumor characteristics at different anatomical sites and time intervals.

## Challenges and future directions

**Fig. 3:**
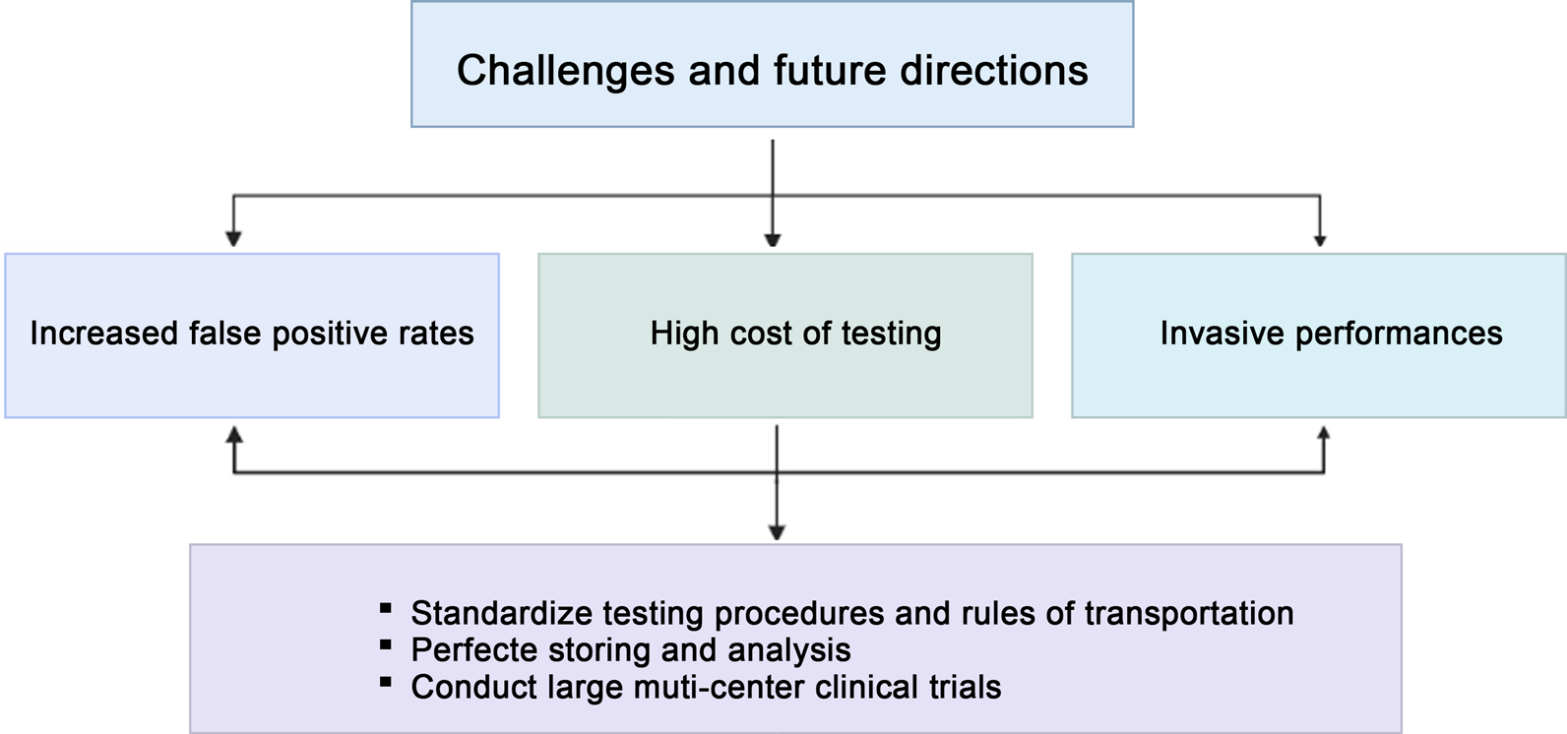
Challenges and future directions of non-blood-based liquid biopsy in the application of breast cancer

Recently, advancements of cell separation and gene detection techniques largely facilitate the development of liquid biopsy. Liquid biopsy has been determined as an important approach in early detection and consecutive monitoring of various malignant disease, including BC. As compared with traditional tissue sample biopsy, liquid biopsy possess unique advantages to better overcome tumor heterogeneity and perform more convenient sampling and testing, thus prompting continuous tracking of malignant progress, therapy effectiveness and relapse. The implication of liquid biopsy offers a valid alternative to traditional testing methods. To date, the published data have mostly concentrated on blood-based biomarkers, such as CTC, ctDNA, ctRNA, and etc. Strikingly, emerging evidence has highlighted the abundance and function of these biomarkers from non-blood sources, including urine, saliva, CSF, pleural fluid and etc. Meanwhile, challenges and obstacles still exist in the clinical transformation of non-blood based biomarkers as well as blood-based biomarkers (Fig. 3).

Mounting studies have detected the potential of non-blood biomarkers and blood-based biomarkers, and their characteristics were also used for further comparison. The published data revealed that the sensitivity of ctDNA from non-blood sources exhibited the level that comparable to those obtained from blood [150, 151]. Zuo et al. revealed that PIK3CA mutation profiling in plasma and urinary ctDNA exhibited an agreement of 97.2% compared with the results achieved for tissue samples [114]. These data could offer important complementary information, contributing to the increased level of overall sensitivity. However, it is worth noting that the false positive rates also increased. The usage of liquid biopsy into non-blood and blood samples could offer specific and sensitive information and reveal the characteristics of different anatomical locations. Amanda et al. revealed the abundance of CSF ctDNA in all 24 patients with BCLM, regardless of negative cytology or borderline MRI imaging. As for plasma-based ctDNA, it was only determined in patients with extracranial disease progression or who had previously received whole brain radiotherapy. Strinkingly, the determination of ctDNA elevation was up to 12 weeks before clinical progress. Moreover, the variations of anatomical disease distribution could largely impact on the concentration of ctDNA. The identification of the sources of genetic information might help to uncover the alterations of anatomical disease and master the whole picture, thus mitigating the effects caused by anatomical alterations. Moreover, evidence showed that the level of plasma ctDNA exhibited no significant alterations in patients with disease progression. Meanwhile, higher concentration of urine-derived ctDNA was identified several months prior to clinical progression [152].

At the present, the progress of techniques, platform and high cost of testing bring obstacles and difficulties in the translation of non-blood and blood-based biomarkers into the clinic. It is known that the usage of different technical methods and platforms could lead to diverse sensitivity and specificity. Moreover, ctDNA is determined to present a short half-life, approximately 2 h, which requires convenient sampling, processing and testing [153]. The addition of stabilizing agent could obtain the extended time to 48 h for the presence of plasma ctDNA, without affecting the testing sensitivity. However, the impact of protective agent on non-blood based markers have not been well-investigated. The invasive performances could add sampling difficulties and increase the uncertainties of sample collection. Besides, it is of great challenging to isolate CTCs from biological fluids, even with the implication of various markers associated with proliferation activities or supplementation with various antibodies. As for BC patients with distant metastasis, CTC isolation becomes much more hard since its emergence are less than extracranial metastasis. The detection of EVs harbouring DNA, RNA, protein, and various metabolites may represent the risk of recurrence as compared with patients with no determination of these markers. Currently, it can help to final assessment in conjunction with other factors. Importantly, standardized testing procedures and rules of transportation, storing and analysis should also be developed and perfected. Further, a large muti-center clinical trials are in an urgent need to testify the clinical practice of liquid biopsies.

Overall, liquid biopsy opens a new avenue for early diagnosis and treatment, especially in cases which samples can not be easily accessed. The deep exploration of circulating tumor biomarkers from non-blood sources could provide new clues to assess the disease progression, treatment response, thus making more accurate and timely strategy. Although advances have been achieved in liquid biopsy, no absolute non-blood based biomarker is determined to be available which was currently used in the clinical management of BC. Much more emphasize should be attached on the development of effective markers, which posses the characteristics of high sensitivity and specificity. The technological advancements are of great necessity to lay a foundation for clinical implementation. In addition, the formulation of standardized methodology of sampling, collection, storage, processing and transportation should be constructed to improve and open up new prospects of non-blood biomarker.

## Data Availability

Not applicable.

## Abbreviations

CTC: Circulating tumor cell
ctDNA: Circulating tumor DNA
ctRNA: Circulating tumor RNA
BC: Breast cancer
CRC: Colorectal cancer
CSF: Cerebrospinal fluid
BrM: Brain metastasis
BCLM: Breast cancer leptomeningeal metastases
FISH: Fluorescent in situ hybridization
CISH: Chromogenic in situ hybridization
EBC: Early breast cancer
MRD: Minimal residual disease
NGS: Next-generation sequencing
CNS: Central nervous system
DdPCR: Droplet digital PCR
WES: Whole exome sequencing
AUC: Area under the curve
EVs: Exosomes are extracellular vesicles
LncRNA: Long noncoding RNA
MMP-1: matrix metalloproteinase-1
MPE: Malignant pleural effusion
SFa: Soluble Fas
MBC: Metastatic breast cancer
TNBC: Triple-negative breast cancer
HPBC: Hormone receptor-positive breast cancer
MPE: Malignant pleural effusion
SCFAs: Short-chain fatty acids
BEAMing: Beads, emulsion, amplification, and magnetics
TAm-Seq: Tagged-amplicon deep sequencing
CAPP-Seq: Cancer personalized profiling by deep sequencing
CAPP-Seq: Whole genome bisulfite sequencing
WGBS-Seq: Whole exome sequencing
WGS: Whole genome sequencing
